# Abstracts and Gleanings

**Published:** 1888-07

**Authors:** 


### ABSTRACTS AND GLEANINGS.

**Tracheotomy in Morphine Poisoning.**—About four months ago I read, in the *Review*, a short account of the resuscitation of a physician of Vienna from opium narcosis by means of tracheotomy and forced respiration with a bellows. It seemed to me to be a rational and feasible procedure, and I determined to try it, should the opportunity present itself

On the afternoon of March 11, 1888, a young man was brought to the City Hospital in an unconscious condition. It was reported that about an hour previously, in ending up a debauch, he had taken an ounce of laudanum with suicidal intent.

His condition then was bad; cyanosis was marked, the pulse proportionately weak; and respiration, already shallow, was rendered difficult by the accumulating mucus in the trachea. The pupils were minutely contracted and immobile; extremities cold. The treatment usually carried out in the hospital in such cases was adopted; one hundredth of a grain of atropia and several syringefuls of whisky were administered subcutaneously, the syphon-tube was passed into the stomach and that organ was repeatedly washed out, at first with water, afterward with strong coffee. The flagging respiration was stimulated by douches of cold and hot water alternately dashed over his chest, and to the same end the faradic current for a time seemed to be of benefit. But, notwithstanding our efforts, narcosis became more profound; cyanosis was intensified to a degree which I have seldom seen, and efforts at respiration on his part ceased entirely, so that artificial respiration was substituted, effectually at first, with much less success afterward. It became evident that unless something radical were done—and that too immediately—the patient could not last. And I bethought me of the bellows method. The patient was hastily removed to the amphitheater, where, with the kind permission of our superintendent, Dr. H. C. Dalton, I performed tracheotomy as rapidly as possible, during which only a gasp was taken now and then, probably two or three to the minute. On separating the severed cricoid, a deep inspiration was followed, as is usually the case at this stage of a tracheotomy, by a considerable interval of quietude. We were about to insert the tube connected with the bellows when a second gasp produced such a shock on the bronchi by the direct impact of cold air on their mucous surface that violent coughing was set up, expelling with each spasmodic expiration mucus which had collected in the trachea to a considerable amount. By this means that tube was soon cleared of its contents. Coincident with the violent coughing, of course, deep inspirations were taken—just the object aimed at, though attained in an unexpected manner, without the use of the bellows; change for the better began almost immediately. The dark purple countenance gradually paled under the more vigorous action of the heart—however paradoxical that may appear, at first thought—and efforts to speak evidenced returning consciousness. A piece of moist gauze placed over the tube acted as a filter to the inspired air. Injections of stimulants—whisky and ether—were continued at intervals, and another hundredth of a grain of atropia was given, after which the patient was removed to his bed and subjected to frequent and vigorous stirring up when respiration was inclined to flag—and it was so inclined for the next several hours. Sleep was not prevented, and he was soon wrapped in its soothing embrace

On the following morning the tube was withdrawn and the incised membrane and cartilage were sutured, the rest of the wound being allowed to granulate.

I should like to be able to close the record of this case *a la mode*, with the statement that recovery followed without a bad symptom, but I am prevented from doing that by the fact that four days after his entrance into the hospital the patient became subject to dilirium tremens, from wh ch he died thirty-six hours later. The presence of pneumonia or other complication was definitely excluded by *post-mortem* examination.

In searching for literature on the subject, the *Index Medicus* directed me to only one article referring to it, that of Dr. G E. Fell, in the *Buffalo Medical and Surgical Journal* for November, 1887. In it the author reports the successful treatment by means of forced respiration with bellows, etc , of a patient who had been poisoned by morphine for a longer time than the one to which I have called your attention. The narcotism in the former case seems to have pursued a course not so rapid as that of the latter. The apparatus used was the one usually employed in the doctor's physiological laboratory in the performance of artificial respiration on dogs. The operation was done on July 24, 1887, prior to the one performed at Vienna, and was therefore, so far as known, the first on record. Since then, Dr. Fell has used the treatment with success in two cases, both of which required the prolonged exercise of forced respiration.

In view of the results of the hospital case I believe that in morphine poisoning, where other means fail, even though it be impossible, on account of lack of apparatus, to supplement it with bellows respiration, tracheotomy is a wise and justifiable measure.—*Dr. Bransford Lewis*, *Weekly Medical Review.*

**Local Application of Calomel in Phagedena.**—I had a case of phagedenic ulceration of the under surface of the glans penis under my charge at the Station Hospital, Brighton, in August last, which defied the recognized treatments of this disease. I applied nitric acid in the most thorough manner on six different occasions during a period of eighteen days without success. I then applied pure carbolic acid, but the disease again returned. Constitutional treatment with opium was adopted throughout. For six days the patient sat in a hot-water hip bath on an average about four hours daily without any appreciable effect on the course of the disease. The condition of the penis on the twenty-first day was as fllows:

A large ulcer existed, covering the entire under surface of the glans, molding it like the mouthpiece of a flute, and extending to the reflected foreskin in the vicinity of the ulcer. A third of the glans had been destroyed. The surface of the ulcer was covered with a reddish gray secretion, irregularly disposed, and pierced here and there by large red granulations. The edges were angry and undermined.

I applied calomel powder on the twenty-first day of the disease, spreading it thickly, and pressing it well into the interstices of the ulcer. The calomel acted like magic; the ulcer began to heal rapidly. Now and then a suspicious spot appeared, but it was at once dissipated by a thorough application of the calomel. The patient made an excellent recovery, and was very pleased at the result, for he believed he was going to lose the whole affair. I could give him very little hope. I had used all the recognized methods of treatment, and the literature of the subject pointed to those slow, creeping ulcerations as almost incurable, except by amputation, and then very often the disease returned in the stump. I was tempted to use calomel, as I have found it very useful in all forms of syphilitic ulceration.—*T. J. Gallwey, British Medical Journal.*

**Intubation of the Larynx.**—Herr Thiersch read a paper at Congress of German Surgical Society, on "Intubation of the Larynx," after O'Dwyer's method. He reviewed the early efforts of Bouchut in this direction, efforts which were disapproved by the Paris Academy of Science of Medicine and had been forgotten, and then referred to the rediscovery of the method in America. At first, he was not inclined to give much credence to the reports of wonderful results obtained by intubation, but after reading the papers presented at a meeting of the New York Academy of Medicine in June last, he had come to the conclusion that the method was of great value, and had determined to put it to a practical test. He had performed intubation in thirty-two cases of diphtheritic croup occurring in the Leipzig Hospital. Of these cases the dyspnœa was permanently relieved in fourteen, of which three recovered. In eighteen cases there was a return of the dyspnœa, and he was obliged to resort to tracheotomy. All of these cases terminated fatally. He attributed the poor results to the fact that the disease was of a very malignant type. In all the cases the immediate result of the introduction of the tube was an instant relief of dyspnœa, but, as before mentioned, this relief was only temporary in about one half of the cases. He thought intubation was specially indicated in cases in which the membrane was limited in extent, and which there was not much swelling of the epiglottis. The inconveniences of the operation were that the almost constant presence of the physician was necessary, because of the accidents which were liable to occur at any moment, and that the presence of an open tube, only partially covered by the epiglottis, exposed the child to greater risk of aspiration pneumonia (Schluckpneumonie). The tube caused ulceration at times.

Herr Rhen had performed the operation fourteen times, with four recoveries. In four other cases he had subsequently resorted to tracheotomy, but without saving the lives of the little patients. In but one of these cases was the child able to swallow well. In some cases he removed the tube several times, once or twice a day, in order to give the patient nourishment.

Herr Rose was a strong advocate of tracheotomy, and had performed the operation 1,926 times for diphtheria, with a mortality-rate of 71.6 per cent.—*New York Medical Record.*

**Intestinal Obstruction Relieved by Laparotomy.**—Dr. G. E. Williamson, New Castle-on-Tyne, in *British Medical Journal*, says: The patient, a groom, æt. twenty two, was first seen in consultation by the operator August 27, 1886. History—no present nor previous hernia, but two attacks of severe abdominal pain two or three years before. August 13, after a large dinner of pork, hastily consumed, he was suddenly seized with severe pain in belly and with vomiting. Next day, after a dose of castor oil, vomiting ceased, but returned in two days, and was fæcal on August 19. On the 21st patient was almost in a state of collapse; pulse small and quick, tongue dry, temperature not raised, fæcal vomiting, abdomen tender about umbilicus and cæcum, no tumor perceptible; no information by rectal examination. August 22 a large enema of fat mutton broth was traced to pass into the cæcum. In two or three hours it was returned without fæcal matter. Temporary cessation of vomiting with improved pulse followed this. It returned, however, and on the 25th an examination of the abdomen was made under chloroform without result. On the 27th, in a fortnight after onset, at the consultation, there was less prostration than was expected—perhaps owing to belladonna and morphine taken—tongue very foul, fæcal odor of breath. Abdomen tense, somewhat tender, and with coils of intestine apparent through wall, resonant throughout except over a small area in right iliac fossa. Abdominal section was decided upon. Only a small quantity of urine was withdrawn by the catheter, although for some days difficulty in passing water had been complained of. As obstruction was thought to be near cæcum, an incision was made in right linea semilunaris, and the peritoneum opened. Hemorrhage extremely slight. Fingers, then whole hand introduced. Nothing found near cæcum. In umbilical region a cord of thickness of a lead pencil passed from behind umbilicus toward pelvis. This when brought to the surface was found to be collapsed intestine. When traced it passed downwads into ordinary gut and upwards into a region of localized peritonitis with adhesions. Two firm bands were brought into view and divided between ligatures, and then adhesions were freed. The wound was sewn up with silk worm gut stitches passed through the whole thickness of the abdominal wall. No spray was used. The surrounding skin was washed with weak corrosive lotion and the sponge wrung out of the same. The bowels acted well a few hours after the operation. In twenty-four hours temperature was 99°; pulse 100. And in every way the *recovery* was rapid and complete, the wound healing in a fortnight under a dressing of

Gamgee tissue. Eight months after the operation the patient was well, no bulging of the scar and no intestinal disturbance. He was doing light work, wearing a belt and broad pad. The strangulation was believed to have been caused either by a twist or by bands secondary to adhesion of the small intestine to the anterior abdominal wall.—*British Medical Journal.*

**Sulphur in Chlorosis.**—In some cases treatment stimulates the secretory activity of the gastric mucous membrane; in others, ferruginous drugs are successful; in others, both proceedings are useless. In these latter cases, there is a deficiency, not of iron, but of sulphur, without which living albumen and active cellular substances cannot exist. Basing on these theoretical considerations, Schulz and Strubing have given sulphur in chlorosis. From the six cases thus treated they draw the following conclusions:

1. In cases of simple chlorosis, in which iron has no effect, the general condition is markedly improved by sulphur.
2. After sulphur has been given for some time, treatment with iron could be started and continued successfully.
3. Sulphur is not borne in cases of chlorosis complicated with catarrhal, inflammatory conditions of digestive tract.

℞. Sulph. depur..................................grs. cl,
Sacch. lact..................................grs. ccc.

M. F. pulv. Half a teaspoonful three times daily.—*Medical News.*

**Salicylate of Mercury in Syphilis.**—Dr. Aranjo, of Rio de Janeiro, gives the following resume of his use of the salicylate of mercury in the *Bulletin de Therap.*, Feb. 29, 1888.

1. Salicylate of mercury is well borne by the stomach. The gastralgias, enteralgias and diarrhœas, which are produced by the other preparations, not excepting the protiodide, do not occur when this preparation is used.
2. In the dose indicated (25 milligrams), stomatitis is never produced.
3. Its internal use acts more promptly and more energetically than other preparations of the same base.
4. Externally, it presents the great edvantages of causing rapid cicatrization of mucous patches and all ulcerative processes. It furthermore causes re-absorption of non-ulcerating syphilomata (papules, tubercles, gummata).
5. In parasitic dermatoses (eczema marginatum of Hebra, circinate pityriasis of Vidal, parasitic sycosis, pityriasis versicolor, tinea favosa and tinea tonsurans) the salicylate of mercury offers the advantages over other preparations of being without odor and non-irritant when the strength is proportionate to the nature of the affection.
6. The salicylate is efficacious in the most inveterate forms of syphilis, and the author believes it will soon replace the protoiodide, the bichloride and the tannate.
7. In the treatment of lepra, it has given very encouraging results associated with gynocardic acid (active principle of the oil of *gynocardia odorata* or chaulmoogra).

8. It has given excellent results in acute and chronic blenorrhagia.

The author knows many colleagues in Rio who have also used this preparation, and speak well of it.—*Maryland Medical Journal.*

**Antifebrin.**—In regard to the method and time of action. The reports referred to show a gratifying uniformity. Temperature begins to fall in about an hour after administration, continues falling for three hours or more, the pulse at the same time diminishing in rate and increasing its tension. Perspiration generally attends the fall in temperature. There is often thirst, and very generally diuresis. The apyrexia persists four to ten hours, quiet sleep generally attending it. The amount of temperature remission is two to seven degrees. The pulse-rate often falls twenty to thirty beats. The increased tension noted is in marked contrast to action of antipyrin and allied drugs. Cahn and Hepp gave sphygmographic tracings, conclusively establishing increased blood pressure, high tension pulse, under the use of antifebrin. The same point has been established in experiments with the drug on animals in Schmiedebourg's laboratory, and it was found that even when injected into the veins antifebrin did not lower blood pressure. This point can not be too much urged as an advantage in the action of antifebrin over antipyrin, since it shows that where antipyrin depresses and weakens the heart's action and the circulation and so becomes unsafe in many cases where it would otherwise be valuable, antifebrin stimulates and stregthens the heart and circulation, and so is beneficial in such cases, aside from its apyretic action. Thus a pulse weak and rapid from disease becomes slow, full and regular when antifebrin is given, an action closely suggesting that of digitalis, which is still further carried out in the *diuresis* so generally resulting.

Adding to this many incidental advantages observed, freedom from vomiting (even in those in whom antipyrin caused nausea or vomiting) no irritation of urinary passages, but on the contrary disappearance of albuminuria in some cases, during its use; clear head and feeling of comfort on the part of the patient, and considering its effectiveness and exceptional safety, antifebrin seems superior to any other antipyretic drug yet discovered.

In the ten cases of rheumatism the dose given was 0.5–1, the daily amount 3–4., followed in all cases by complete disappearance of swelling and pain in three to five days. Four of these cases relapsed, three having received less antifebrin than the other cases, the fourth, a very severe case, and only given a total amount of 12.5.

The report closes with the claim for antifebrin in acute articular rheumatism of "complete cure without the disagreeable incidental effects of salicylic acid."—*Boston Medical and Surgical Journal.*

[Care should be exercised in the administration of this most valuable antipyretic and a second dose should not be given until we learn of the patients temperature, this should be borne in mind as by the repeated giving of this remedy we may reduce the temperature below the normal and get some very disagreeable symptoms.—ED. *Abstract and News.*]

**Influence of Medicines taken by Nursing Women Upon their Infants.**—If salicylate of sodium be taken by the nurse in doses of 1 to 3 grammes, the author has found that it may be recovered in the urine of the child. The same is true in respect of iodide of potassium.

Ferrocyanide of potash given in doses of 1 to 2 grammes for three doses was not recovered from the urine of the infant.

If iodoform is given to the nurse by external application, will be traced three or four days afterwards in the urine of the infants, as iodine. In none of the cases in which these experiments were tried did any harm result to the infant. Elimination of mercury by means of the milk rarely occurs.

If citric acid in a 1 to 90 solution be given for four days, the quantity of the solution not exceeding 3 grammes, and if as much as 7½ grammes, of a solution of hydrochloric acid, 5 to 180, be administered to a nursing woman in the course of four days, the author's experiments show that the milk undergoes no perceptible change, and the child will experience no harm. The author concludes that acid food, salads, for example, need not be withheld from nursing women, at least so far as their acidity is concerned.

Tincture of opium given to nursing women in doses of 25 gtts., resulted in neither drowsiness nor constipation on the part of the infant. Morphine given hypodermically in doses of 2·8 milligrammes resulted very seldom in disadvantage to the infant, chloral produced bad effects somewhat more frequently, especially if the hypodermic injection were given a very short time before the child was put to the breast. Atropine given in doses of 1 to 5 milligrammes was in no case followed by dilatation of the child's pupils. The author believes that fever in the mother is not sufficient cause for weaning her child, but the child should be weaned if the mother has erysipelas or scarlatina.—*Journal de Medicine.*

**The Treatment of Mastitis.**—Dr. J. S. Westerfield, in *Practioner and News*, writes of a case of mastitis: The gland was indurated, painful, and discharging from three openings. She suffered from nervousness, emaciation, and loss of sleep, had no appetite; and was taking opiates freely. I applied the rubber plaster as directed in Prof. Yandell's article, after introducing horse hair for drainage. Results, immediate relief from pain and return of appetite. She took no more opiates, slept well the first night, and in two weeks the plaster came off; the case was discharged, being about well.

In July, 1887, Mrs. G., mother of several children, the last one three months old, had a large abscess of right breast, which had been discharging for two or three weeks. She told me she would prefer to die at once rather than live and suffer another week as she had been doing for a week past.

Washed out cavity with antiseptic solution and applied rubber plaster. Result, early relief from pain and anxiety. The next day she was cheerful and expressed herself as feeling well. In ten days the plaster came off and she was discharged, being well.

In January, 1888, Mrs. McC., mother of three children, the last one six months old, called at my office. An abscess had a month before formed in the left breast, discharged and healed, but left an induration the size of a goose egg. At the time of her visit the gland had again inflamed and was intensely painful. I detected no sign of fluctuation, but it was my opinion that pus could be reached by cutting deep.

I did not cut, however, but applied rubber plaster; and she sent me word several days later t at I had entirely cured her abscess and that she suffered but little pain after the plaster was put on.

January 23, 1888, I visited Mrs B., mother of one child, still nursing at the age of fifteen months. I found an abscess of the left mammary gland that should have been opened a week before. Cut abscess, and let out at least one pint of as an offensive pus as I ever smelled. The treatment this time consisted of antiseptic washes and support of the gland by bandage, together with an iron tonic. I saw the case again on February 10th, and learned that a neighboring physician had again lanced the breast a week prior to my second visit. At this time both openings were discharging Pus was detected in another part of the gland and evacuated. I washed out cavities with a solution of carbolic acid and applied plaster. I have since learned that the cure was complete.—*American Practitioner and News.*

**Bacilli of Intermittent Fever.**—This specimen is exhibited through the kindness of Dr. William T. Councilman, associate professor of pathology in the Johns Hopkins University. It presents the organisms first described by Lavaran. They are contained in the blood corpuscles that are abstracted from the body of he patient just before, during or after the chill characteristic of intermittent fever. They are always present at that time, being contained in numerous corpuscles, and are not found except at that period—about the time of the chill. Nor are they found in the blood of individuals who are not suffering from intermittent fever. Of course this does not prove any casual relation of the organisms to the disease; that requires cultivation outside the body and innoculation with pure cultures. These have not been made; therefore it would not be justafiable to say—and observers do not say—that these are the cause of intermittent fever. But observations are sufficiently numerous to warrant the assertion that these organisms are characteristic of the blood of patients suffering from that disease. In that respect they rank with the spirillum of Obermeier, which was one of the earliest organisms observed in the blood.

Dr. N. S. Davis: Did I understand that they are found only in the blood at the stage of chill—after the chill disappears, during the febrile stage?

Dr. Belfield: That is the universal statement of observers. They are present for a short time before and after the chill.

Dr. R. H. Babcock: Has the efiect of quinine been tried on these organisms?

Dr. Belfield: Not to my knowledge, in the blood outside of the body. The administration of quinine to the patient, however, causes a rapid and complete disappearance of these bodies from the blood corpuscles.—*Peora Medical Monthly.*

**Uterine Hemorrhage in Pregnancy.**—*Parish.*—Case of hemorrhage from the uterus in a woman eight months pregnant. Whether a case of placenta previa or not, Dr. Parish said that the proper treatment here was to put the woman to bed and keep her there, and not allow her to rise from it for any purpose whatever. He advises a physician who has a case of placenta previa or suspected placenta previa on hand, to provide himself with a Barne's dilator. In a dangerous hemorrhage, this will not only dilate the os for delivery, but will also act as a tampon.

It is not well to keep a dilator in the office as you keep other instruments, because the rubber loses its elasticity in about two months, and is then useless.

If you have no dilator, use the tampon; though of course only when absolutely necessary. He does not approve of absorbent cotton for tamponing, as recommended by Parvin; for he says that the cotton, on account of its great attraction for fluids, is likely to favor the hemorrhage rather than to check it.

For his own part, he prefers a long strip of muslin or linen, such as an ordinary roller bandage, soaked in bi chloride. Especial care should be taken that the material is tightly packed around the os; then the vagina is to be filled; and finally external pressure kept up by a T-bandage.

If indelivery it be necessary to perform version, give an anæsthetic, in order to relax the uterus, and thus avoid the laceration of it, otherwise almost certain.

After delivery, hypodermic injections into the uterus of hot water, or even a styptic app ied to the internal surface of the uterus, will stop the bleeding if the inertia of the uterus is too great for proper co ntraction.—*Medical Times.*

**Diphtheria Carried by Turkeys**—Dr. Paulinis, in the *Bulletin Medical*, reports a most interesting epidemic of diphtheria which occurred in Skiatos, one of the Grecian isles, in the year 1884. The population of this island at the time was about four thousand. Dr. Bild, an old practitioner, is the authority for the statement that for thirty years no case of diphtheria had been known on the island. In June a child aged twelve years was attacked with diphtheria, and died. Seven other cases occurred in the immediate neighborhood: five of these died. The disease extended, until, within a period of five months, one hundred persons were attacked, of which number thirty-six died. Three weeks before the sickness of the first child, a flock of turkeys arrived from Salonica. Two of these were sick on arrival, and each of the others was subsequently attacked. Dr. Paulinis found in the throats of the sick ones patches of false membrane. The glads of the neck were swollen, and in one bird the disease had extended to the larynx, making it hoarse, One of the turkeys, after recovery, had paralysis of the legs, and was unable to walk. Although there had been no immediate contact between the sick birds and the first child attacked, still the distance between them was slight, and a wind had been for some time blowing in a direction favorable to the transportation of the disease. Dr. Paulinis believed that the disease was contracted from the turkeys, its germs being carried by the currents of air.—*Science.*

**Creoline.**—This novel antiseptic is a new product unknown out of Germany. We know only that it is obtained by the distillation of pit coal, but its preparation and composition will be new questions to be unravelled by our chemists. It is a deep brown liquid of syrupy consistence, having the odor of tar, and giving with water a milky emulsion.

Creoline has a more powerful action than phœnic acid on the bacilli of cholera, typhoid fever, and the staphylococcus pyogenes aureus. It is also a powerful disinfectant, for the odor of putrid liquids is immediately destroyed by the addition of 1-1000, while the addition of phœnic acid gives no result. The same disinfecting results were obtained with the powder, and a creoline soap.

In solution of 1 or 2 per cent. the best effects are produced for tamponment of wounds; it replaces advantageously iodoform, and more, possesses very excellent hemostatic properties in the dressing of wounds and ulcers. It is in no degree toxic.

The following are some of the applications and dosage: 1 per cent. solution for gonorrhœa (cured in four days). In leucorrhœa good results have been experienced. In many cases of burns and frost bites creoline diminishes the suppuration and pain, and dries the wound. In affections of the conjunctiva, creoline in the form of pomade produces rapid amelioration. In three patients having opacity of the cornea, it seems to have caused the disappearance of the opacity. In one case of grave diphtheria, a local application of 5 per cent. produced in five days very considerable amelioration.—*Journal de Med. de Paris.*

**Jaborandi in Obstetric Practice.**—Dr. Jerome Hardcastle, in *Medical and Surgical reporter*, says:

Having for many years noted the fact that parturition does not progress favorably till *diaphoresis* occurs, I have for some months past induced this condition, in the early stage of labor, by giving fl. ext. jaborandi (green—the brown has proved worthless in my hands). My plan is, when called to a case, to order a warm brick to be applied to the feet—which are always cold, and then to give one-third of a teaspoonful of fl. ext. jaborandi in half a wineglassful of water, and repeat the dose every half-hour until perspiration occurs. It is very seldom that more than two doses are required. The first effect of this medicine on the patient is soothing, she becomes more quiet, and bears her pains with resignation. Upon being questioned the patient often states that her pains do not hurt her as they did. On examination, after diaphoresis occurs, the os will be found dilating rapidly; the soft parts to be in a favorable condition; and in a short time the labor will be satisfactorily terminated. Should the patient appear weak from the sweating, I wipe her face and neck with a dry towel, and give her a teaspoonful of whiskey or half as much of aromatic spirits of ammonia.

Since using the above remedy, I have had no occasion to use ether, chloroform, or the forceps. I have not seen any mention of the use of jaborandi in obstetric practice; but having had such favorable results from its employment, I recommend it to the consideration of the profession.

**The Treatment of Tonsilitis by Salicylate of Sodium.**—Dr. A. Hillaby writes in the *London Practitioner* for April, 1888, that with the desirability of having some really reliable remedy for tonsillitis and the fact that this disease bears some not distant relationship to acute rheumatism, has led him to try the salicylate of sodium in every case during the past four years. The action of the drug has come up to his best expectations; the fever which in some cases reached 103° was speedily reduced, the action of the skin promoted, the resolution of the inflammation in the throat hastened and the formation of tonsillar abscess averted.

His plan of treatment is as follows:

Open the bowels freely with a good dose of mistura sennæ co., put the patient on milk diet and administer the following draught:

| | | |
|---|---|---|
| ℞. | Sodii salicylatis .......................... | gr. x–xv. |
| | Tincturæ aurantii corticis .................... | *m* x, |
| | Aqu., ad.... ............................ | ℥ j. |

M. To be taken every four hours.

When the inflammation in the throat begins to subside, the dose of the salicylate of sodium is reduced and is given in smaller doses for a few days after all throat symptoms have disappeared—*Md. Medical Journal.*

**The doctors** (Seward and Goodman) who claim to have discovered gleditschine (stenocarpine) as an anæsthetic and mydriatic alkaloid of *gleditschia tricanthos*, announce that they are ready to stand by their statements.

There may be such an alkaloid and it may possess the properties claimed for it, but there can be no doubt about the solutions examined by Parke, Davis & Co., being simply cocaine and sulphate of atropine.—*Pacific Record.*

**Haya Poison; Confirmation of its Anæsthetic Powers.**—Dr. F. Goldsmidt, of Nurenberg, after careful trials of the Haya poison, concludes that though it will have a less extensive field of application than cocaine, it is in some respects more useful, because it does not produce the effects on the pupil noticed with the latter, nor does it affect accommodation and introcular pressure. He found a 1 per mil solution produced perfect anæsthesia in from ten to fifteen minutes, so that the connective tissue and cornea could be treated even with the actual cautery without pain. Though the effect only lasted three or four hours, the anæsthesia could be prolonged at will by the daily administration of a small dose. Slight but temporary conjunctival irritation seems to have been present at first in most cases, which was worse if the eye was already inflamed. The anæsthesia was accompanied by vascular dilatation, not as with cocaine by contraction. Experience can alone determine whether it will be available for deeper operations.—*Centr. f. Klin. Med.*

**Inter-Cranial Neuralgias or Migraine.**—These are either direct or reflex. They are explained by the meningeal, cerebral or ganglionic nerves which diffuse themselves in the meninges and penetrate the cerebrum, following the direction of the vessels which they embrace in their plexuses.

Migraine is at times accompanied by vomiting and a hypersensibility of the eye and ear. It demands absolute repose, washing of the intestinal canal by the seidlitz salt, and the internal use of aconite and caffeine (one granule of each every fifteen minutes until sedation. If the migraine is due to periodic cause, it should be combatted by quinine (arseniate or hydroferrocyanate, one granule every fifteen minutes during the paroxysm).

If the cause is chlor-anemia, iron arseniate should be used (one granule four to six times a day in the interval of paroxysms. Aconitine and caffeine being used during the access).—*Western Medical Reporter.*

**The clergy** have lately become much concerned over the future of physicians. Sam Jones says he would not care to go to heaven if he thought there were any doctors there. [The doctors have yet to be heard from—ED.] He doesn't know how it is that the study and practice of medicine makes men irreligious. In his experience it has been a rare thing for him to meet a religious doctor. At the late commencement exercises of the Detroit Medical College of Medicine, the clergyman who made the address, also expressed the belief that there are no doctors "over there." He, however, was not ungracious enough to ascribe their absence to their wickedness, but simply to the fact that there are no sick angels. It did not seem to occur to our reverend brother that physicians could take part or pleasure in the exercises of the place, but that, as is the case here below, they must be doctors or nothing.—*Medical Age.*

[The Rev. Sam. would quickly cry out for a doctor if severe sickness or accident should overtake him.—ED. RECORD.]

**Cocaine in the Vomiting of Pregnancy.**—In the vomiting of pregnancy cocaine has been used by many observers; the experience of Weiss and Bois has been previously noticed in these reports, the former giving the drug by mouth every half hour in sixteenth-grain doses in solution; the latter using a two-per-cent admixture with vaseline, and placing a tampon smeared therewith against the cervix morning and evening. Fraipont has reported several successful cases, and has employed the drug by subcutaneous injection (20 minims of a four-per-cent. solution) into the epigastrium. Engelmann reports an obstinate case in which he was successful in two days, giving thrice daily by mouth ten-minim doses of a ten-per-cent. solution. Phillips has used the drug in two cases, once with a marked success; in his second case it was uncertain whether the cessation of vomiting could be fairly attributed to the use of cocaine.—*American Practitioner and News.*

**Communicability of Disease from Animals to Man.**—The transmission from the cow to man of scarlet fever and tuberculosis was the subject of the opening address of Prof. Hamilton at Marischal College, Aberdeen, in which the lecturer gave an excellent account of the investigations conducted by Mr. Power and Dr. Klein into the relation of a cow malady to scarlet fever in man. He referred also to the observations of Capland, who believed that both the dog and the horse could suffer from the latter affection, and stated that a febrile condition of some kind can be commuicated to animals by innoculating them with the blood of persons who are the subjects of scarlet fever. He further expressed the opinion that tubucle could be conveyed to man by means of milk from tuberculous cows. While the possibility of such ocurrence cannot be denied, it must be borne in the mind that Klein has pointed out that there are certain important differences between bovine and human tuberculosis; and again, Creighton has shown that man occasionally suffers from a form of this disease which resembles the bovine malady, making it probable that by far the greater number of cases are not of bovine origin. Nevertheless, the subject deserves much greater investigation, and certainly every effort should be made to prevent the distribution of milk from tuberculous cows.

**Oil of Mullein in Enuresis.**—We have recently noticed several reports on the subject of mullein oil and its use as a remedy for enuresis, one an obstinate case of nocturnal enuresis, that had resisted the most strenuous preventive measures, rhus aromatica, scutellaria, santonine, belladonna, and numerous other agents, where the effect of the oil of mullein was prompt and satisfactory. A writer in a western medical journal says that it is not probable that it will prove a specific in every case, but it should be added to the list as an agent liable to do good work in time of need.

A practitioner from Lynn, Mass., says, "I have treated many cases of enuresis, mostly nocturnal, some of which had resisted years of treatment, both old school and new, and I do not know of one thus treated that has not been cured."

The doses will vary somewhat with the caprice of the prescriber. We would suggest a dilution of the oil in alcohol—one part of oil to fifty or a hundred parts of alcohol. Of this give 5 or 10 drops at a dose, four or five times daily.—*Med. Summary.*

**Dysmenorrhœa** in its most violent forms has been relieved by Meniere, by giving an enema consisting of bromide of potassium aud chloral, 30 grains of each; one-half of this amount to young girls.—*American Practitioner and News.*

**Reflex Neuroses Due to Constipation.**—Besides the affections of the circulatory system, and especially owing to palpitation, irregular pulse, and vertigo, which should be attributed to the stimulus of the cardiac branch of the great sympathetic, Kisch believes that rebellious hemicrania often have no other cause than constipation. Certain kinds of sciatic neuralgia, of lumbar-abdominal pains, ovarian crises, and even neuralgias of the fifth pair, may be due to habitual constipation. Although Kisch has succeeded in forty-eight cases where the cessation of constipation was followed by the cure of various chronic neuralgias, we may ask ourselves whether intestinal sluggishness was indeed a sufficient cause for these various neuralgias. The variation of this condition may bring about different results.—*Journal des Sciences Medical de Lille.*

**Wart-Chaming Extraordinary.**—A well known and old established firm of chemists in Birmingham, England, is doing a lucrative trade in charming away warts. The *modus operandi* is somewhat novel. The principal partner in the business gravely examines the warts, counts them, and instructs the patient to depart and return in a week's time. The order is obeyed, and when the individual makes his appearance on the second occasion, the chemist places on each wart a piece of bacon which he has prepared in the interim, indulges in a solemn incantation, tells the patient he must not thank him, and quickly walks away. The warts are said to wither away in the course of a few days afterwards.—*Chemist and Druggist*, Feb. 4, 1888.

**Acute Rheumatism.**—Dr. C. B. McClay says of the treatment of acute rheumatism (*Peoria Medical Monthly*, April, '88): As to treatment, we may well say that every imaginable form of prescription has been used. Heat and cold, acid and alkaline, wet and dry, catharsis and astringency and the salicylic treatment, all about to the same effect. I have seen the best results by first results from the plan of—1. Relieving pain by morphia internally and anodyne applications externally; 2. Bromide potass. and Dover's powder as a special nerve tonic; 3. Iodide potass. and hydaochloride amonium for alterative, perspirative and diuretic, according to the indications. But to arrest the irritatbility, overcome the pain is the main issue, for then the perspiration becomes normal and the patient convalesces.

**A New Elastic Mucilage.**—The *Chemische Centralblatt* gives the following directions for an elastic mucilage, which differs from the formulæ in general use:

Dissolver 1 part of salicylic acid in 20 parts of alcohol, add 3 parts of soft soap and 3 parts of glycerine. Shake thoroughly and add the mixture to a mucilage prepared from 93 parts of gum arabic and the requisite amount of water (about 180 parts).

This mucilage is said to keep well, and when it dries, to remain elastic without tendency to cracking. It will prove valuable if it possesses all of these qualities.—*National Druggist.*

